# Targeting age-related inflammation in myelodysplastic syndromes

**DOI:** 10.18632/oncotarget.26266

**Published:** 2018-10-23

**Authors:** Yang Mei, Peng Ji

**Affiliations:** Yang Mei: Department of Pathology, Feinberg School of Medicine, Northwestern University, Chicago, IL USA; Peng Ji: Department of Pathology, Feinberg School of Medicine, Northwestern University, Chicago, IL USA

**Keywords:** myelodysplastic syndromes, innate immune response, ageing, microenvironment, damage associated molecular pattern

Myelodysplastic syndromes (MDS) are a group of clonal hematologic malignancies characterized by cytopenia, ineffective hematopoiesis, and an increased risk of progression to acute myeloid leukemia (AML). MDS is phenotypically and genetically heterogenous. The most common cytogenetic abnormality in MDS is heterozygous loss of chromosome 5q [del(5q)] [1].

Accumulating evidence reveals that dysregulation of the innate immune signaling is a featured signature of MDS with del(5q). Many genes that are involved in the innate immune signaling are located in or in close proximity to the common deleted regions of chromosome 5q, including *DIAPH1,* MiR-146a*, TIFAB*, have been extensively investigated recently. Loss of *miR-146a* in mice leads to dysplastic megakaryocytes by derepression of its downstream target-tumor necrosis factor receptor-associated factor-6 (TRAF6) [2]. Overexpression of TRAF6 in mouse bone marrow mimics the features of MDS through IL-6 over-secretion [3]. *DIAPH1* encodes mDia1 that is involved in linear actin polymerization. Our previous report revealed that mDia1 heterozygous and knockout mice develop MDS phenotypes with age [4]. Mechanistically, these mice aberrantly overexpress CD14 on granulocytes in a cell-autonomous manner, which in turn leads to a hypersensitive innate immune response to lipopolysaccharide (LPS) stimuli through CD14/Toll-like receptor 4 (TLR4) signaling. In patients with del(5q) MDS, *DIAPH1* is also decreased with corresponding increase of CD14 on granulocytes. More recently, TRAF6-interacting protein-TIFAB, another haploinsufficient gene in del(5q), was found to reduce TRAF6 protein level via a lysosome-dependent degradation [5]. TIFAB deficient cells show dysregulation of innate immune-related signature with a hypersensitivity to TLR4 stimulation, which contributes to ineffective hematopoiesis.

**Figure 1:**
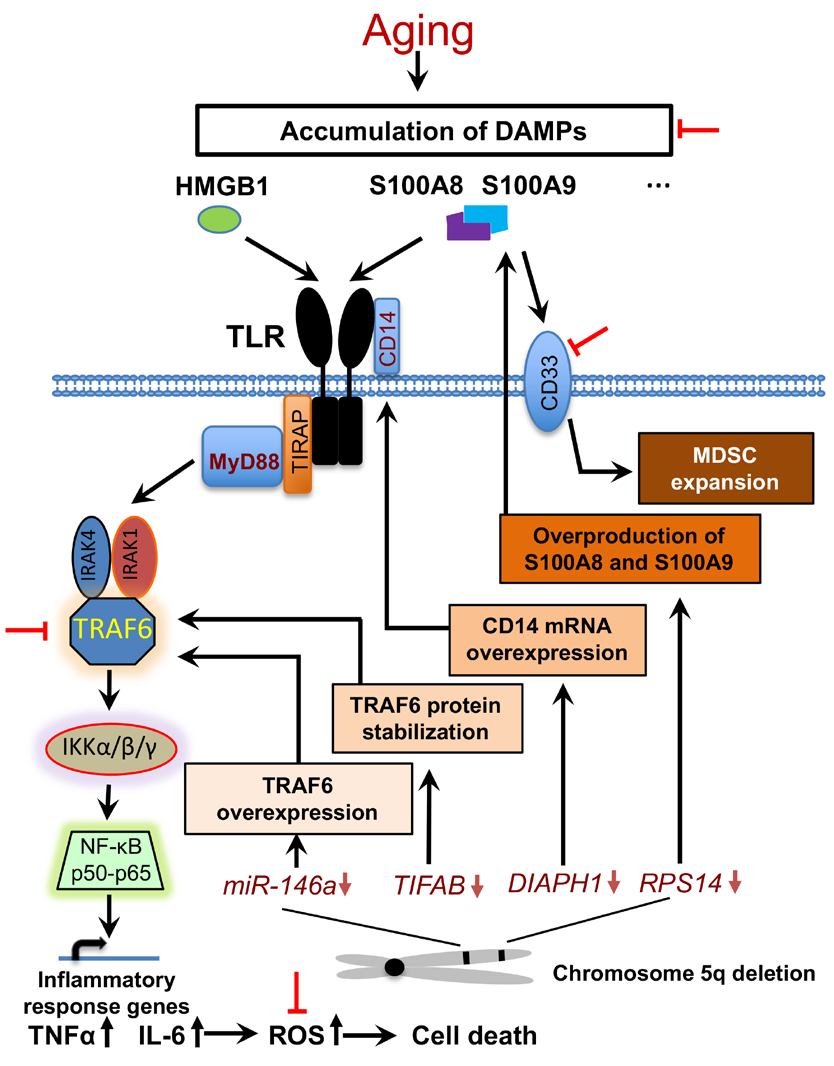
Aberrant Innate Immune Signaling Pathways in del(5q) MDS During aging, DAMPs are gradually accumulated in the bone marrow microenvironment and trigger Toll-like receptor (TLR)-mediated innate immune response. Haploinsufficiency of several 5q genes, including MiR-146a, *TIFAB*, *DIAPH1* and *RPS14,* induce an aberrant activation of the innate immune signaling and overproduction of pro-inflammatory cytokines through different mechanisms. Cell death caused by cytokine-mediated reactive oxygen species (ROS) generates more DAMPs. MDSC expansion, which is achieved through S100A9-CD33 ligation, results in an increased number of effective cells engaged in the innate immune signaling pathways. The potential targetable molecules are highlighted with red inhibition symbols.

It was more recently reported that haploinsufficiency of ribosomal protein Rps14, another 5q gene, leads to p53 activation and subsequent erythroid differentiation defect [6]. This differentiation blockade is mediated by increased expression of host damage-associated molecular pattern (DAMP) proteins including S100A8 and S100A9. As an endogenous TLR4 ligand, S100A8-S100A9 heterodimer promotes the activation of the transcription factor NF-κB and the secretion of pro-inflammatory cytokines such as TNFα and IL-6. Overall, these studies define the unique contributions, but complex means of alterations in the innate immune signaling and inflammation in the pathogenesis of MDS (Figure 1).

MDS is primarily an age-related neoplasm. Dysregulation of the innate immune signaling and inflammation is closely associated with the aging process. To better understand the relationship between inflammation, aging, and the pathogenesis of MDS, we generated a mouse model with dual deficiency of mDia1 and miR-146a (DKO). These mice develop age-related anemia and ineffective erythropoiesis that closely mimic human MDS [7]. To reveal whether these MDS phenotypes are cell-autonomous due to dual deficiency of mDia1/miR-146a or induced by the microenvironment, we transplanted the bone marrow cells from DKO mice into young (2-month-old) or old (1-year-old) recipient wild type mice. The young recipient mice started to develop MDS phenotypes when they were 7 to 8 months old. Old recipient mice did not recover from transplantation-related anemia. These results demonstrate that the old bone marrow microenvironment is critical for the development of MDS. Further study reveals that pro-inflammatory cytokines, especially TNFα and IL-6, are significantly elevated in DKO mice. These cytokines are mainly generated by myeloid-derived suppressor cells (MDSCs). *In vitro* challenge of MDSCs from DKO mice with DAMPs triggers robust induction of TNFα and IL-6 with up to nearly 600-fold on the mRNA level of IL-6. We also demonstrated that pathologic levels of TNFα and IL-6 inhibit erythroid colony formation and differentially affect terminal erythropoiesis through reactive oxygen species (ROS)-induced cell death [7].

Importantly, gene expression profiles of the cultured and cytokine-treated bone marrow erythroblasts and the erythroblasts from DKO mice share the same inflammation pathway as one of the most important signaling pathways [7]. Therefore, our study further highlights the significance of multiple factors in the innate immune signaling in the pathogenesis of MDS. It also underscores the cooperative roles of the aging bone marrow microenvironment and genetic abnormalities in the development of ineffective erythropoiesis in MDS.

Immunosuppressive therapy has been well established to treat a variety of neoplasms and blood-related diseases. However, therapeutic approach targeting immune dysregulation in MDS shows only modest efficacy. The complex and heterogeneous nature of MDS could be one of the major reasons. Nevertheless, Lenalidomide, an agent for the treatment of low risk del(5q) MDS, has been shown to exert immunomodulatory effects partially through inhibiting the production of pro-inflammatory cytokines TNFα and IL-6 [8]. On the other hand, direct inhibition of cytokine production, such as anti-TNF and anti-IL-6 therapies, was shown to have less efficacy in response rate and transfusion reduction in unselected, low rick MDS patients in several clinical trials [9]. Although the results of these studies are disappointing, novel approaches such as combined therapy or targeted treatment of specific subgroup(s) of patients would be future directions to pursue. In addition, activation of TLR signaling cascade in del(5q) MDS makes the Toll-like receptor and(or) the adaptor protein CD14, as well as the downstream effectors, such as IRAK (Interleukin-1 receptor-associated kinase) and TRAF6, to be attractive therapeutic targets (Figure 1). More recently, innovative effort targeting MDSCs in del(5q) MDS through engineered CD33 antibody, which blocks S100A9 binding availability with its receptor, significantly improved hematopoiesis *in vitro* [10]. The area of immunotherapy in MDS is rapidly evolving. With our increased understanding of the aberrant innate immune responses in the pathogenesis of MDS, novel approaches, including the clearance of DAMP and(or) ROS in the aging microenvironment, may change our future management strategies in MDS.
